# Tongue shape classification based on IF-RCNet

**DOI:** 10.1038/s41598-025-91823-1

**Published:** 2025-03-01

**Authors:** Tiantian Liang, Haowei Wang, Wei Yao, Qi Yang

**Affiliations:** 1 School of Electrical Engineering, Dalian Jiaotong University, 794 Huanghe Road, Dalian, 116028 China; 2 Department of Traditional Chinese Medicine, The Second Affiliated Hospital of Dalian Medical University, Dalian, 116000 Liaoning China

**Keywords:** Deep learning, Tongue diagnosis, Mixed input, Feature fusion, Medical research, Preclinical research, Information technology

## Abstract

The classification of tongue shapes is essential for objective tongue diagnoses. However, the accuracy of classification is influenced by numerous factors. First, considerable differences exist between individuals with the same tongue shape. Second, the lips interfere with tongue shape classification. Additionally, small datasets make it difficult to conduct network training. To address these issues, this study builds a two-level nested tongue segmentation and tongue image classification network named IF-RCNet based on feature fusion and mixed input methods. In IF-RCNet, RCA-UNet is used to segment the tongue body, and RCA-Net is used to classify the tongue shape. The feature fusion strategy can enhance the network’s ability to extract tongue features, and the mixed input can expand the data input of RCA-Net. The experimental results show that tongue shape classification based on IF-RCNet outperforms many other classification networks (VGG 16, ResNet 18, AlexNet, ViT and MobileNetv4). The method can accurately classify tongues despite the negative effects of differences between homogeneous tongue shapes and the misclassification of normal versus bulgy tongues due to lip interference. The method exhibited better performance on a small dataset of tongues, thereby enhancing the accuracy of tongue shape classification and providing a new approach for tongue shape classification.

## Introduction

**Fig. 1:**
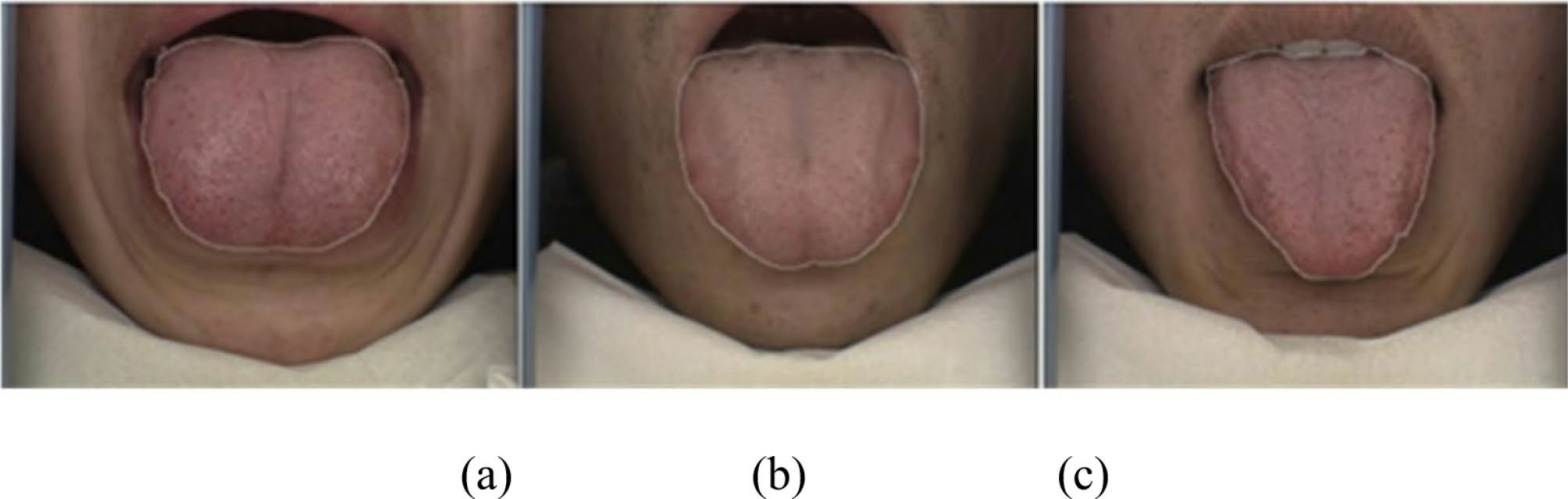
Classification of tongue shape (**a**) bulgy tongue (**b**) normal tongue (**c**) thin tongue.

Clinical diagnoses in traditional Chinese medicine (TCM) are based on the clinical practice of doctors; after thousands of years of accumulated diagnostic experience, it has been theorized that diseases can be diagnosed on the basis of a patient’s external characteristics. Tongue diagnosis is an important diagnostic approach in TCM^1,2^. By analyzing the links of various tongue-related features, such as tongue shape, tongue coating, tooth-marked tongue, and tongue color, with internal diseases of the human body^3^, a TCM-based approach to tongue diagnosis that deduces the relevant diseases on the basis of the differences exhibited by tongue features has been developed. The size and geometric shape of the tongue (e.g., round, ellipsoid, hammer-shaped, or U-shaped) are important tongue features^4^ and are closely related to the patient’s age and health condition. By observing the changes in tongue shape in patients of a certain age, it is possible to diagnose whether a patient is suffering from a certain disease. Therefore, the study of tongue shape is highly practical. However, TCM-based tongue diagnosis relies heavily on the personal experience and subjective judgement of TCM doctors, which can lead to misdiagnosis and reduce diagnostic efficiency when facing complex tongue features. Figure 1shows three types of tongue shapes that have certain similarities in appearance and are prone to manual misdiagnosis. In recent years, medical diagnosis based on artificial intelligence (AI) assistance has gradually become a popular research topic with the development of artificial intelligence research^5^. Researchers have applied intelligent image processing technology to TCM-based tongue diagnosis^6^ to improve objectivity and diagnostic efficiency and reduce doctor workload in TCM tongue diagnosis. There has been some progress in research on tongue-related diagnoses, but some problems remain to be solved.

Owing to the multiple difficulties in performing quantitative and qualitative analyses of tongue features, the current stage of tongue diagnostic research focuses on tongue segmentation and classification tasks.

Tongue segmentation aims to extract the tongue body in a complex background environment to provide a basis for subsequent tongue diagnosis. Early research on tongue segmentation focused on traditional image processing methods based on edge detection, region growing and mixed processing^7–10^; however, the segmentation effect of such methods is sometimes unsatisfactory when dealing with problems such as tongue color similarity or blurring of tongue edge contours. In recent years, image segmentation methods based on deep learning have been widely used for tongue segmentation and have achieved superior performance compared with that of traditional image segmentation methods because deep learning methods can accurately distinguish the differences between pixels when processing images^11^. Zhou et al.^12^ combined deep learning methods to propose an end-to-end network model called TongueNet for tongue localization and segmentation multitask learning. Huang et al.^13^ proposed an improved UNet model for tongue image segmentation by combining a residual soft join module and a salient image fusion module to address the effects of complex open environments and device factors. To address the interference of different tongue shapes and textures on tongue segmentation, Cai et al.^14^ built TSRNet on the basis of encoder and decoder frameworks and by combining a priori knowledge and a patch segmentation module to accurately segment tongues with different shapes and textures. Song et al.^15^ proposed RAFF-Net, a network for automatic tongue segmentation, which was constructed via a residual architecture and a multiscale feature fusion module on a UNet-based backbone network. This construction approach improved the network’s ability to perceive the tongue by improving the training loss function. Experimental comparisons have revealed that the segmentation precision of RAFF-Net was greater than that of traditional UNet. The above study shows that the network architecture with encoder and decoder structures is currently a popular research direction for tongue segmentation, and UNet has shown extraordinary performance in the tongue body segmentation task.

Tongue images include various features, such as color, tooth-marked, coating, and shape, and tongue feature classification is the key direction of current research on tongue diagnosis. Tongue feature classification via AI can assist doctors in objectively recognizing complex tongue features. For tongue color classification, Hou et al.^16^ used a convolutional neural network to classify tongue color, and Ni et al.^17^ proposed TongueCaps by combining CapsNet and a residual block structure to achieve end-to-end tongue color classification. Tooth marks have received increasing attention as more common and relatively obvious tongue features. For example, Li et al.^18^ proposed the use of a three-stage approach to classify the dentate tongue. In the first stage, a candidate region is extracted via the first neural network layer. In the second stage, deep features are extracted via a convolutional neural network. In the third stage, an accurate classification is performed via a multi-instance support vector machine (MI-SVM). Tang et al.^19^ proposed a two-stage classification strategy by introducing a cascaded convolutional neural network for detecting tongue regions and feature points, which are used for final classification via a fine-grained classification network. Because tongue mark labeling and region annotation require considerable work, which is not conducive to clinical applications, Zhou et al.^20^ proposed an end-to-end deep neural network, i.e., the weakly supervised tooth-mark detection network (WSTDN), which only needs to annotate tooth-marked or non-tooth-marked tongue maps and can be used for tooth-marked tongue recognition on the basis of weakly supervised learning. As tongue coatings have certain color and shape features, tongue coating classification has received increasing attention. To classify tongue coatings accurately, Tang et al.^21^ first used a priori knowledge to obtain suspicious tongue coating patches; then, they used tongue coating features extracted by a convolutional neural network; finally, they used an MI-SVM to classify the extracted tongue coating features. To reduce the workload of processing tongue coating features, Okawa et al.^22^ proposed YOLOv2 as a tongue coating feature region extraction network by calculating the tongue coating index (TCI) for each region, using transfer learning on the ResNet-18 network, and finally completing the tongue coating classification task by calculating the TCI for each region.

Because similar tongue shapes have a certain degree of variability from one body to another, accurately categorizing tongue shapes is difficult. Most of the current methods for tongue shape classification still focus on traditional image processing methods. For example, Huang et al.^23^proposed a geometric feature defined by the length, area and angle measurements of the tongue to classify tongue shape. Zhang and Zhang^24^ used a computerized method to perform a quantitative analysis combined with a decision tree model to classify tongue shapes. Considering the prominence of deep learning methods in tongue feature classification, Huang et al.^25^ built a tongue segmentation classification network (TSC-WNet) model based on a feature fusion approach, which combines the tongue segmentation results with the original inputs for tongue shape classification. However, considering tongue shape uncertainty, the effectiveness and practicality of the above methods need to be investigated further.

A single tongue segmentation task only involves tongue body extraction from the image and cannot analyze the features contained in the tongue body, thus making it difficult to provide efficient help for diagnosis. Most existing tongue feature classification tasks are completed by processing the global image information. Additionally, in the complex background environment of the tongue body, some similar interfering features are easily misperceived, which results in misclassification. Multiple interfering features contained in the complex background environment also require a greater number of network layers and more feature extraction computations, which lead to network overfitting and reduce network training speed. Tongue segmentation can extract the tongue from a complex background environment, and the feature fusion method can enhance the feature extraction ability of the network without increasing the number of network layers. Therefore, using the feature fusion method to combine tongue segmentation and image classification tasks can aid in recognizing tongue shapes in complex background environments. Herein, we propose a tongue-shaped classification network, IF-RCNet, which consists of a two-level structure of a segmentation network named RCA-UNet and a classification network named RCA-Net. Tongue shape classification is accomplished by feature fusion in the form of nesting the segmentation and classification networks, which can be accomplished with small samples and can accurately classify some of the different tongue shapes that have similar features.

Therefore, in this paper, IF-RCNet, a deep learning network based on feature fusion, is applied for tongue body segmentation and classification. The remainder of this paper consists of three parts. The “Methods” section describes tongue shape recognition based on IF-RCNet. The “Results” section describes the details of the experiment and reports the quantitative and qualitative outcomes. The experimental results are discussed in the “Discussion” section. Conclusions are drawn in the “Conclusion” section.

## Methods

**Fig. 2:**
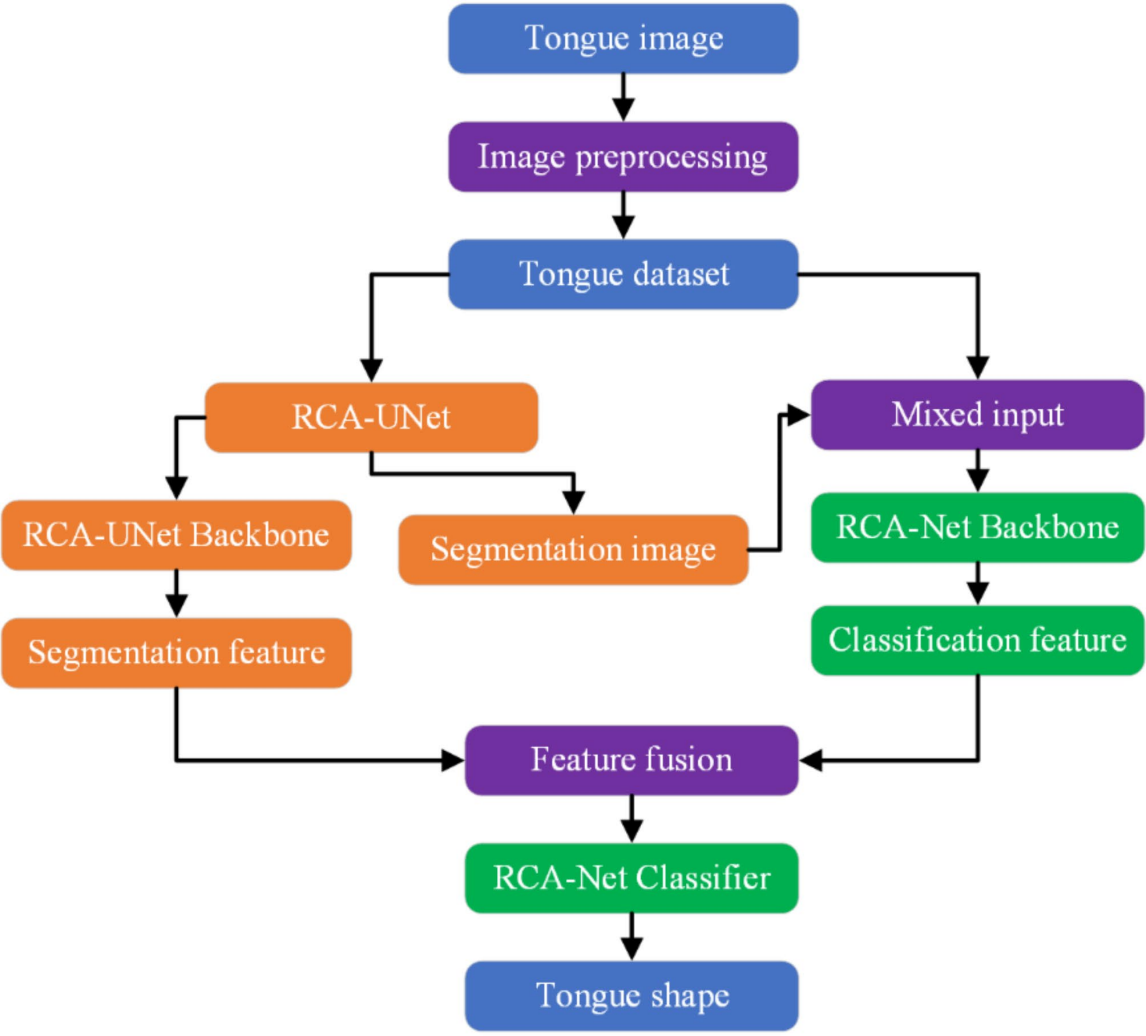
Flow of tongue shape recognition based on IF-RCNet.

In this section, detailed explanations of the data collection and preprocessing, network construction, training, and evaluation of IF-RCNet are provided. The specific implementation flowchart is shown in Fig. 2. First, the captured tongue images undergo preprocessing to create a dataset for network training. The tongue image data are input into both the RCA-Net and RCA-UNet networks. After processing via RCA-UNet, the segmented tongue and learned tongue features are obtained. Next, the segmented tongue images are combined with the original inputs of RCA-Net, and the tongue features are processed via feature fusion operations to become part of the backbone network parameters of RCA-Net, thereby forming the IF-RCNet, which is used for tongue shape classification. Finally, the network parameters are continuously optimized on the basis of the model evaluation results to enhance the performance of IF-RCNet. The detailed implementation process is presented in the following sections.

### Dataset construction and preprocessing

**Fig. 3:**
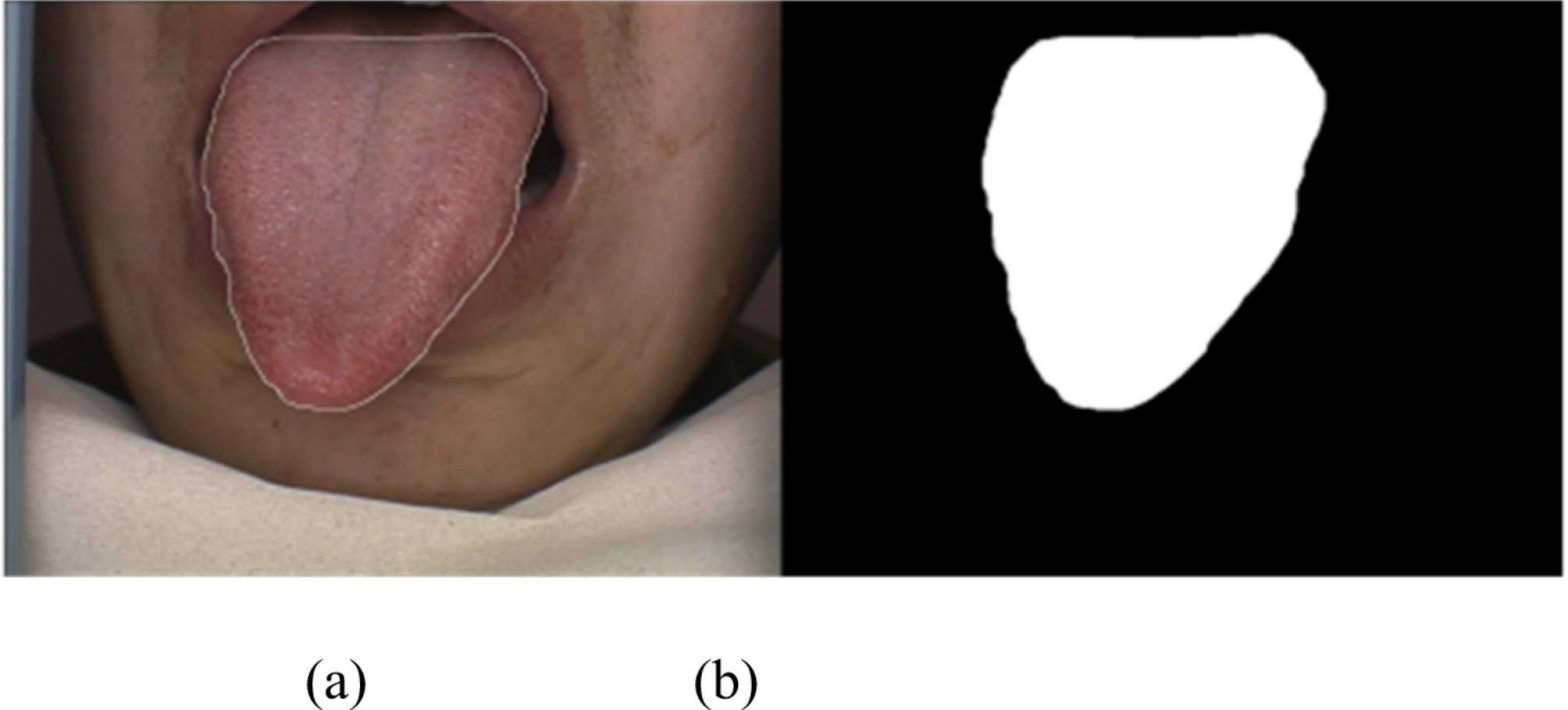
Tongue body labeling. (**a**) original tongue body image and (**b**) tongue body with background labeling image.

Considering that the subsequent network training and evaluation require a relatively stable dataset with consistent data forms, we used an EOS RP professional camera manufactured by Canon Japan and acquired tongue images in a well-lit and consistent indoor environment. The processed tongue images were transferred to a computer with a clear display, and three professional Chinese physicians with many years of clinical experience from the Second Affiliated Hospital of Dalian Medical University labeled the tongue regions and classified the shapes. Then, the processed images were made into a standard image dataset. The specific labeling results are shown in Fig. 3.

Moreover, the publicly available tongue image dataset BioHit Tongue is introduced for network training and evaluation. This dataset contains a total of 300 tongue photos, and each image is annotated via a segmentation mask. The dataset was processed by a TCM doctor to remove 95 images, making it difficult to discriminate the tongue shape, and the selected tongue images were classified. Table 1 shows the total data volume of the two datasets and the data volume of each tongue shape.

**Table 1:** Number of images of the three tongue shapes in the two datasets.

Datasets	Thin	Bulgy	Normal	Total
Our	253	331	226	800
BioHit	58	49	98	205

A cross-validation method is introduced to evaluate the performance of the network model and solve the problem of the limited data in the two datasets. First, the dataset is divided into a training set, a test set and an evaluation set according to ratios of 3.5, 0.5 and 1, respectively. During the training process, a different subset is selected as the test set each time, and the rest is used as the training set and evaluation set. The process is repeated five times. Finally, the five performance evaluation results are averaged to obtain the evaluation results. Moreover, the format of the images is unified to 224 × 224 pixels to reduce the overall network parameters and reduce the network training time.

*Ethics statement.* The data collection and processing methods used in this study were conducted in accordance with guidelines and regulations. This study was approved by the ethics committee of the Second Affiliated Hospital of Dalian Medical University. All participants were informed of the purpose of the study and their privacy was protected before the start of the study. Informed consent was obtained from all individual participants included in the study.

## Construction of tongue shape classification based on IF-RCNet

**Fig. 4:**
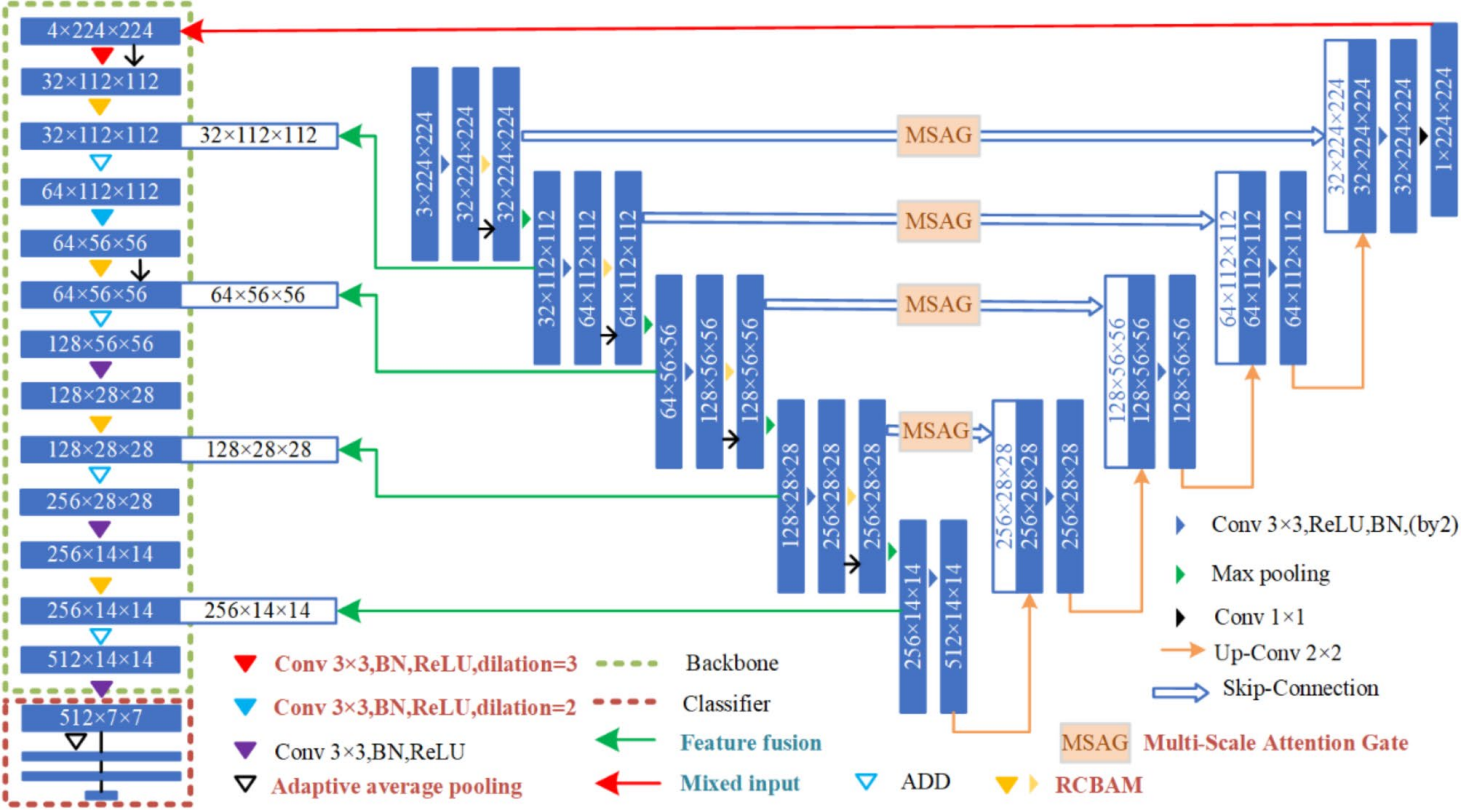
Overall framework of IF-RCNet. The two dashed boxes represent RCA-Net, whereas the remaining portion on the right represents RCA-UNet. The light blue font represents improvements in data processing, whereas the dark red font represents improvements in network structure.

The performance of deep learning network models is affected by a variety of factors, including network depth, structure and input data quality. Therefore, we propose a tongue shape classification network with a two-level structure nested with tongue segmentation and tongue shape classification (shown in Fig. 4 to improve classification network accuracy and reduce the dependence on data sample quality). By using mixed input and feature fusion, the segmentation mask of the segmentation network is mixed with the original input as the classification network input. The features extracted from the segmentation and classification networks are fused to accomplish tongue shape classification. The network can complete model training on a small tongue image dataset with a strong ability to perceive complex features and high classification accuracy. To ensure the quality of the input data and make full use of the data, cross-validation is used for network training, and the average of the results from five training runs is taken as the final output.

*Classification network* To obtain high tongue shape classification accuracy, a classification network including an improved backbone and improved network classifier, namely, RCA-Net, is proposed, and its overall network structure is shown in the left half of Fig. 4. On the one hand, the improved backbone is proposed on the basis of a convolutional neural network structure containing five convolutional blocks, all of which are composed of a 3 × 3 convolution, a batch normalization (BN) layer and a rectified linear unit (ReLU) activation function. In particular, in all five convolutional blocks, the first two convolutional layers are replaced by dilated convolutions with a larger receptive field, and a residual convolutional block attention module (RCBAM) that embeds into all the convolutional layers is proposed. On the other hand, the improved network’s classifier focuses mainly on structural improvement.

First, common approaches that add pooling operations to increase the receptive field often result in the loss of some details and smaller targets, leading to decreases in the network’s learning ability. By enlarging the learning scope of the pixel points, dilated convolution can increase the receptive field while keeping the input feature dimensions unchanged, thereby enhancing the network’s feature extraction capability. Therefore, the first two convolutions in the network are designed as dilated convolutions with dilation factors of 3 and 2, which learn the target information from the 6 × 6 and 5 × 5 pixel regions, respectively, to allow the network to learn shallow features.

**Fig. 5:**
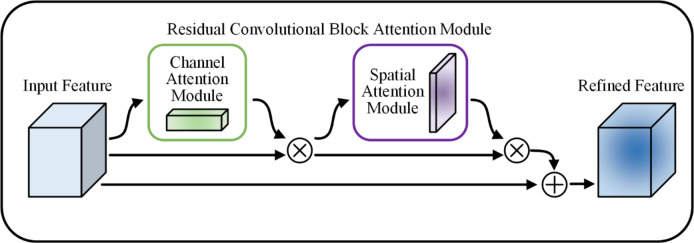
RCBAM with a residual architecture.

Furthermore, considering that the background environment of the tongue is more complex and that the network will lose part of the feature information during downsampling, the network’s ability to learn complex features needs to be enhanced. The attention mechanism processes the information in the feature map, selects important information vectors, derives the weights of the target feature information on the basis of their relevance, and effectively improves the ability to learn complex features. Moreover, by compensating for the output end with the input, the residual architecture can effectively compensate for the feature loss caused by pooling operations. After combining the attention mechanism and residual architecture, as shown in Fig. 5, an RCBAM is proposed. Specifically, an RCBAM is an attention module that is based on a residual architecture and is built from two submodules, the channel attention module (CAM) and the spatial attention module (SAM). By multiplying the output features of the CAM and SAM element by element and adding them to the input features of the residual branch to obtain the final output features, this attention module can be used to enhance the feature representation of different channels and extract the key information of different locations in space. Moreover, a branching structure with RCBAM and a maximum pooling operation is added to the backbone to enhance the network’s ability to model and represent the image features and reduce network complexity to some extent.

Finally, to adapt to the feature fusion operation, after the last network convolutional block, an adaptive average pooling operation is used to adjust the pooling size and stride according to the input size, performing average pooling adaptively at each position in the input, and then delivering to a classifier. Considering that a powerful classifier is essential for ensuring the performance of the classification network, an improved network classifier consisting of three fully connected layers is proposed, and the ReLU activation function and dropout layer are added between the two fully connected layers to prevent overfitting in the network.

*Segmentation network* As part of the data input and tongue feature source for the tongue shape classification network RCA-Net, the tongue segmentation network needs to have strong segmentation accuracy and feature extraction capabilities. To achieve this, RCA-UNet is also built by using RCBAM. In addition, the network’s downsampling and skip connection are enhanced by dual convolution and the multiscale attention gate (MSAG), respectively. The overall network structure is shown in the right half of Fig. 4.

First, a dual-structured convolution and maximum pooling layer are used as the network downsampling layer. Downsampling occurs four times, and the same RCBAM as the classification network is added to each downsampling layer. The dual convolution consists of two identical 3 × 3 convolution layers, ReLU activation functions, and BN layers. The first 3 × 3 convolutional layer extracts shallow features such as edges, whereas the second 3 × 3 convolutional layer learns deeper abstract relationships between pixel points. This dual convolution structure helps extract deeper tongue body feature information, improves the network’s ability to extract tongue body features from complex backgrounds, and provides more tongue shape-related information as input to RCA-Net.

**Fig. 6:**
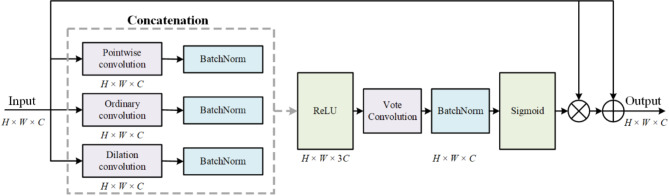
Overall structure of the MSAG. H, W, and C represent the height, the width, and the change of the feature map.

Considering that the UNet network is a convolutional neural network structure, information extraction from higher layers is often partially lost during the downsampling process. Therefore, the skip connections in the network are also strengthened. The MSAG module (shown in Fig. 6) is added to the network skip connection. The first part of the MSAG is composed of pointwise, ordinary, and dilation convolutions. They filter the feature information of the input feature map to complete tongue feature extraction. In the second part, the BN layer performs normalization after splicing, and the ReLU activation function performs activation. The third part, i.e., vote convolution processing, is followed by the output results, and input feature map multiplication is performed. Finally, the multiplication results and the input feature map are summed to obtain the results.

After downsampling and skip connection, network upsampling occurs, which consists of double-structured convolution and deconvolution. The feature map output from the downsampling process is input into the corresponding upsampling module through skip connections. After being processed by the dual convolution, the feature map is upsampled through deconvolution to increase its dimensionality. After four upsampling operations, the feature map is processed by the activation function and restored to a tongue segmentation result map that matches the size of the input image.

## Training the tongue shape classification model

The tongue classification model is trained and evaluated via IF-RCNet in PyTorch 1.9.0 with CUDA 11.1 and cuDNN 8.2.0. We set the batch size to 2 to implement the training for the classification network, use the ReLU as the activation function, and use the Adam algorithm with a learning rate of 0.0001 for optimization. The training process uses stochastic gradient descent (SGD) to achieve faster convergence. For the binary classification problem, the segmentation network classifies only single pixels in the image and uses the cross-entropy loss function as the single-pixel binary classification training strategy. The network is trained via the same training strategy as the classification network. We used three fully connected layers as classifiers for IF-RCNet, AlexNet, VGG16 and ResNet18. The first and second fully connected layers use the same 512 nodes for feature representation; in the third fully connected layer, three nodes are used to accomplish label prediction. The implementation environment was a 64-bit Windows server with hardware comprising an Intel Core i5-12400 F CPU, 64 GB of RAM and an Nvidia RTX 3080Ti GPU.

## Evaluation indicators

The overall performance of the classification network was examined via the accuracy rate and F1 score to complete the test and obtain accurate evaluation results for the quantitative tongue classification network. The specific evaluation formulas for the accuracy rate and F1 score are shown in Formulas (1), (2), (3), and (4).

1 $$\:\text{Precision=}\frac{\text{TP}}{\text{TP+FP}}$$

2 $$\:\text{Recall=}\frac{\text{TP}}{\text{TP+FN}}$$

3 $$\:\text{Accuracy=}\frac{\text{TP+TN}}{\text{TP+TN+TP+FN}}$$

4 $$\:{\text{F}}_{\text{1}}\,\text{s}\text{core}=\frac{\text{2}\times\text{Precision}\times \text{Recall}}{\text{Precision}+\text{Recall}}$$

Tongue segmentation is a type of semantic segmentation, that involves pixel-level classification. We use the mean intersection over union (MIoU), Dice coefficient and mean Hausdorff distance (MHd) for segmentation evaluation to quantitatively analyze the tongue segmentation accuracy. The formulas for the MIoU, Dice coefficient and MHd are shown in (5), (6) and (7).

5 $$\:MIoU=\frac{1}{k+1}\sum\:_{i=0}^{k}\frac{TP}{FN+TP+FP}$$

6 $$\:Dice=\frac{2\times\:TP}{FN+2\times\:TP+FP}$$

7 $$\:{d}_{H}\left(X,Y\right)=\text{m}\text{a}\text{x}\left\{\begin{array}{c}sup\\\:x\in\:X\end{array}\begin{array}{c}inf\\\:y\in\:Y\end{array}\text{d}\right(x,y\left)\begin{array}{c}sup\\\:y\in\:Y\end{array}\begin{array}{c}inf\\\:x\in\:X\end{array}\text{d}\right(x,y\left)\right\}$$

In the above equation, *k* represents the number of all image categories except the image background, *TP* indicates that the predicted samples and the real samples are both positive cases, *FP* indicates that the predicted samples are positive cases and the real samples are negative cases, *TN* indicates that the predicted samples and the real samples are both negative cases, and *FN* indicates that the predicted samples are negative cases and the real samples are positive cases. *x* and *y* denote two sets. *x* and *y* represent the two points in the set. sup(supremum) and inf(infimum) denote the upper and lower bounds of certainty, respectively.

## Results

### Ablation experiment results and analyses

Ablation experiments are conducted on the tongue-shaped classification network via BioHit and a self-constructed dataset to validate the effectiveness of the proposed feature fusion and mixed input methods. The performances of a single tongue classification network, RCA-Net, and three improved networks based on RCA-Net with feature fusion, mixed input, and both feature fusion and mixed input are tested and evaluated. The specific performance is shown in Table 2. The results of the ablation experiments are quantitatively and qualitatively analyzed in this section.

**Table 2:** Evaluation metrics obtained from the ablation experiments performed on the two datasets.

Dataset	Model	Mixed input	Feature fusion	Accuracy	F1 score
Our	RCA-Net			74.75%	73.61%
	RCA-Net + Mixed input	√		75.50%	73.91%
	RCANet + Feature fusion		√	75.75%	74.47%
	RCA-Net + Mixed input + Feature fusion	√	√	76.87%	76.29%
BioHit	RCA-Net			72.19%	70.50%
	RCA-Net + Mixed input	√		77.17%	78.92%
	RCA-Net + Feature fusion		√	75.61%	72.76%
	RCA-Net + Mixed input + Feature fusion	√	√	84.87%	84.22%

To show the effectiveness of the proposed method quantitatively, the ablation experiment results are shown in Table 2, where RCA-Net is used as the baseline model for comparison. As shown in the table, the RCA-Net + mixed input indicates the application of the segmentation results mixed with the original tongue image as the input to the classification network to obtain more tongue feature inputs for expanding the data input to the network. RCA-Net + feature fusion refers to applying the fusion of the tongue feature information extracted from the coding layer of RCA-UNet with the tongue feature information extracted from the backbone network of RCA-Net, thus enhancing the overall quantity of data input to the network. IF-RCNet is a tongue classification model that applies both mixed input and feature fusion. Table 2 shows that using mixed inputs on both datasets resulted in 0.75% and 4.98% increases in accuracy, respectively, as well as 0.3% and 8.42% increases in F1 scores, respectively. Applying feature fusion increased the accuracy by 1.00% and 3.42% and increased the F1 score by 0.86% and 2.16%, respectively. Each of the improvement approaches improves the network performance at different levels. Table 2 shows that IF-RCNet (RCA-Net + Mixed input + Feature fusion) performs best. The accuracies of IF-RCNet are 2.12% and 12.68% greater than those of RCA-Net. In terms of F1 scores, IF-RCNet achieves increases of 2.68% and 13.72% over those of RCA-Net on both datasets. These findings further demonstrate that IF-RCNet can effectively learn more representations of tongue features and can achieve more accurate classification results.

**Fig. 7:**
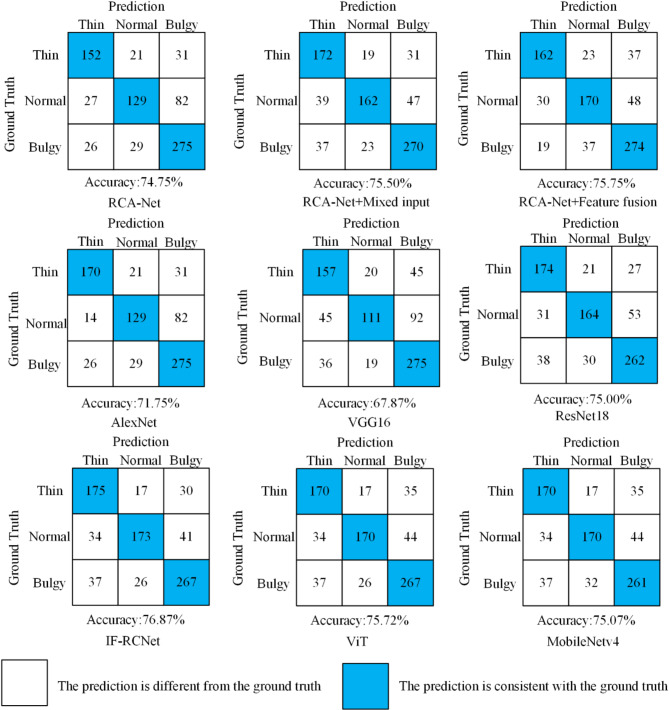
Tongue classification results on the self-constructed dataset.

**Fig. 8:**
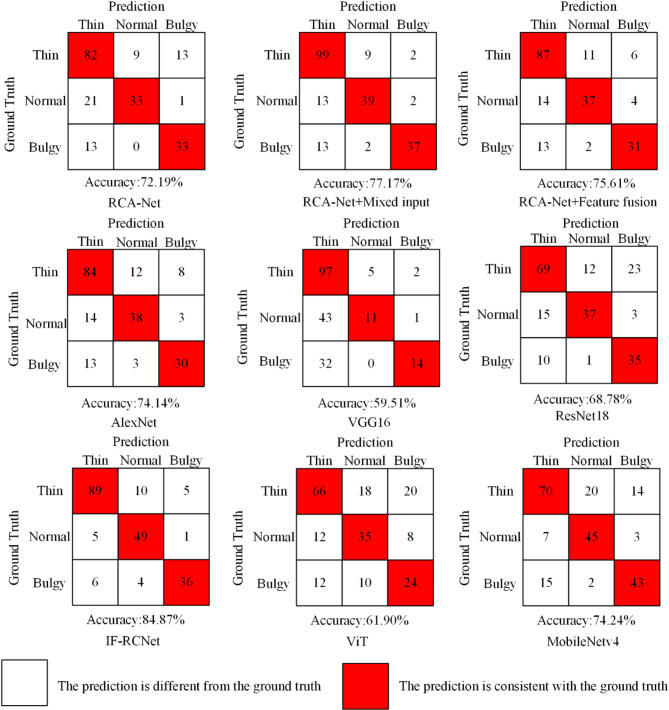
Tongue classification results on the BioHit dataset.

Moreover, the results of qualitative analyses further validate the effectiveness of the proposed method. To visually evaluate the effectiveness of IF-RCNet for tongue shape classification, the classification results of tongue shape in the two datasets are shown in Figs. 7 and 8. The blue part of the figure represents the classification results on our dataset, and the red part represents the classification results on the public dataset BIoHit. Some bulgy tongues have a high degree of similarity in shape to normal tongues, and it is difficult to distinguish them accurately on the basis of the contour appearance of the tongue alone. Additionally, the complex background environment contained in tongue images easily interferes when extracting tongue features. Furthermore, there is some similarity between the lips present around the tongue and the textural features of the tongue body; thus, it is easy to misclassify the tongue that is in contact with the lips as a bulgy tongue in the tongue shape classification. The above problems can be effectively solved by adding mixed input and feature fusion methods. These matrices show that adding input mixing to RCA-Net can effectively improve the network’s overall ability to classify normal tongues; adding feature fusion to RCA-Net can reduce the network’s misclassification of normal and bulgy tongues. Finally, IF-RCNet performed better in terms of recognizing all three tongue shapes compared to the other three networks, i.e., the basic RCA-Net classification network, RCA-Net with a mixed input structure, and RCA-Net with feature fusion.

## Tongue shape classification comparative experiment results and analyses

The comparison models for the experiments are AlexNet, VGG16, ResNet18, Vision Transformer (ViT)^26^and MobileNetv4^27^. Among the five comparison models, the first three are all classical convolutional neural network structures and have the same fully connected layers as IF-RCNet. The fourth comparison model, namely, ViT, is an image classification model based on the transformer architecture, primarily utilizing the self-attention mechanism to extract contextual information from images and using a multilayer perceptron (MLP) for feature extraction and classification. The last model, MobileNetv4^27^, is a lightweight classification network model built upon the improved MobileNet architecture and optimized via neural architecture search (NAS) methods. The results of both quantitative analyses and qualitative analyses are reported herein to fully illustrate the effectiveness of the proposed method.

**Table 3:** Evaluation results obtained from comparison experiments of multiple models on two datasets.

Dataset	Model	Accuracy	F1 score
Our	AlexNet	71.75%	70.10%
	VGG 16	67.87%	63.95%
	ResNet 18	75.00%	73.37%
	ViT	75.72%	74.65%
	Mobilenetv4	75.07%	73.22%
	IF-RCNet	76.87%	76.29%
BioHit	AlexNet	74.14%	72.50%
	VGG 16	59.51%	38.90%
	ResNet 18	68.78%	67.53%
	ViT	61.90%	41.11%
	Mobilenetv4	74.24%	72.88%
	IF-RCNet	84.87%	84.22%

First, the results of the quantitative analyses are shown below. The specific results of the evaluation of the proposed IF-RCNet and the competitor models on both datasets are shown in Table 3. Obviously, IF-RCNet achieves the best results in terms of accuracy and F1 scores on both datasets, and in our dataset, ResNet-18 exhibits better classification performance than AlexNet and VGG 16, which proves that the residual architecture strategy can improve the overall network accuracy. Moreover, the proposed IF-RCNet has the same residual architecture as ResNet-18, while the classification accuracy is greater than that of ResNet-18, not only because of the residual architecture but also because the other improvements can enhance the performance of the proposed network. Additionally, ViT demonstrates superior performance compared with other network architectures, which proves that the attention mechanism is effective in handling more complex classification tasks. Although RCA-Net enhanced by RCBAM and MSAG lags behind ViT with an MLP, which is mainly limited to the self-performance of convolutional neural networks, it still exhibits excellent performance and benefits from the structural improvement approach of this study. Moreover, the performance of IF-RCNet, which improves upon that of RCA-Net through the introduction of mixed inputs and feature fusion methods, outperforms that of ViT. On the one hand, this improvement is due to the segmentation of the tongue image in the mixed inputs, which removes some of the interference. On the other hand, this improvement is due to the feature fusion method, which expands the number of tongue features in the RCA-Net backbone. Specifically, IF-RCNet resulted in improvements of 5.12% and 6.19% in accuracy and F1 score compared with AlexNet, respectively, and improvements of 9.00% and 12.34% in accuracy and F1 score compared with VGG 16, respectively. Compared with ResNet18, IF-RCNet achieved improvements of 1.87% and 2.92%, respectively. Compared with MobileNetv4, the performance was improved by 1.80% and 3.07%, respectively. Moreover, compared with the most powerful ViT, the accuracy and F1 score were increased by 1.15% and 1.64%, respectively.

In the BioHit Tongue image dataset, the evaluation results of VGG16, ResNet18 and ViT are significantly lower than those on our dataset, mainly because of the influence of the quantity of data in the dataset and because overfitting may occur in networks that are too deep. MobileNetv4 performs similarly on the two datasets, which implies that, specific to this task, its structural improvement has a weak ability to handle insufficient data. In contrast, in this study, the receptive field is enhanced by replacing classic convolution with dilated convolution, which increases the ability of the proposed model to learn the global information. Then, the input data of the classification network are expanded by adding the classification results, which ensures the required information for model training, while also removing the background interference of the classification results, which enhances the backbone network’s ability to focus on target learning. Furthermore, sufficient fusion of the classification network features is implemented, which reduces the complexity of the network structure and avoids overfitting during network training. The proposed IF-RCNet is 10.63% better than MobileNetv4 in terms of accuracy in handling limited data and 11.34% better in terms of F1 scores. The quantitative results fully support the above analyses.

The results of qualitative analyses further confirm the above results. Figures 7 and 8 show that classifying thin and normal tongues is a problem that is easily confused by all networks, mainly because part of the normal tongue has some similarity with the thin tongue in terms of texture and color features. It is also easy to see that some of the normal and thin tongues have the same morphological features because of the influence of individual body mass. Additionally, the contact between the thin tongue and the lips leads to the misclassification of the thin tongue as a bulgy tongue. Nevertheless, IF-RCNet still achieves the highest number of accurate classifications for thin and normal tongues, largely addressing the issue of similarity between thin and normal tongues, as shown in Fig. 7. The main reason is that the feature fusion method provides more detailed information to the RCA-Net backbone, thus allowing the network to capture the subtle differences between normal and thin tongues. Compared with ViT (which performs the second best in terms of accuracy), IF-RCNet better addresses the misclassification issue of thin tongues in contact with the lips. This is mainly due to the mixed inputs, which remove the lips from the segmented tongue images, thereby reducing the impact of complex backgrounds on the learning process.

As shown in Fig. 8, IF-RCNet performs best for classifying normal tongues and outperforms other models in terms of accurately classifying thin and bulgy tongues. Although VGG performs well in classifying thin tongues, it incorrectly classifies many normal tongues as thin, resulting in lower overall accuracy. Its model shows clear overfitting and a poor ability to learn tongue shape features. Moreover, IF-RCNet clearly outperforms MobileNetv4 in accurately classifying thin tongues. This process primarily depends on the mixed input, which expands the input data for RCA-Net and allows the network to learn more about the subtle differences between normal and thin tongues. Additionally, the feature fusion operation also results in more feature information. While retaining as much of the texture information as possible, a single tongue contour that removes the interference information is added, which greatly improves the generalization ability of the network on different datasets and enables the network to achieve excellent classification performance. The qualitative results fully support the above analyses.

Overall, the quantitative and qualitative experimental results and analyses on both datasets fully validate the effectiveness of the proposed IF-RCNet.

## Tongue segmentation comparative experiment results and analyses

**Fig. 9:**
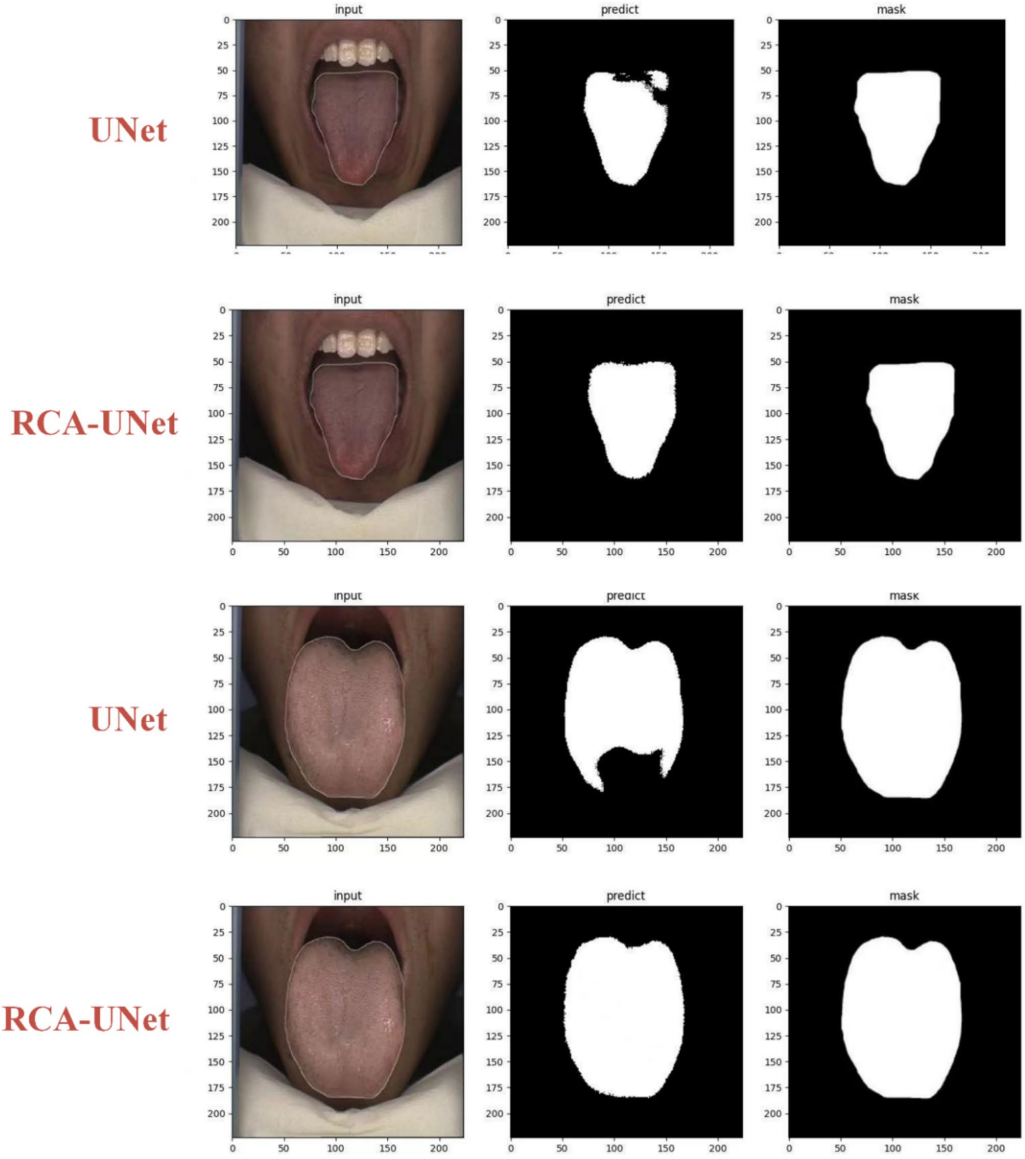
Comparison of tongue segmentation results.

In IF-RCNet, accurate segmentation of the tongue body is important for the mixed input, while the tongue feature extraction capability also has an impact on the subsequent feature fusion. Therefore, the effectiveness of the proposed improved tongue segmentation network RCA-UNet was analyzed on two datasets; the specific evaluation results are shown in Table 4. Moreover, to visualize the performance differences between the networks more intuitively, the segmentation results were qualitatively analyzed, as shown in Fig. 9.

**Table 4:** Shows that the proposed RCA-UNet performs better on the two tongue image datasets. In terms of the MIoU score, RCA-UNet improves by 0.62% on our dataset compared with UNet and improves by 2.44% on the public dataset BioHit. In terms of the dice score, the proposed RCA-UNet outperforms UNet by 0.36% and 1.62%, respectively. Additionally, RCA-UNet decreases by 0.11 and 0.4 on MHd, proving that RCA-UNet segmentation produces results that are more similar to those of the manually labeled mask model. These data indicate that the introduced RCBAM has learned more tongue features, which effectively improves the network’s ability to distinguish between the target and the background. The changes in MHd indicate that RCA-UNet, compared with UNet, retains more information, thus compensating for the feature loss in the network.

Dataset	Model	MIoU	Dice	MHd
Our	UNet	93.08%	96.28%	4.51
	RCA-UNet	93.70%	96.64%	4.40
BioHit	UNet	96.05%	97.61%	3.63
	RCA-UNet	98.49%	99.23%	3.23

Qualitative analyses, which are also presented in this section, illustrate the effectiveness of the improvements of the proposed IF-RCNet in tongue segmentation. By observing the two images in the upper part of Fig. 9, the RCA-UNet with the residual RCBAM structure is shown to have a stronger feature extraction ability when confronted with the shadows contained in the tongue body. Compared with UNet, the RCA-UNet with the residual RCBAM structure achieves accurate segmentation of the tongue’s shadowed areas, which strongly validates the powerful feature extraction ability of RCBAM. When segmenting the input image in the following two images, the tongue body appears to be somewhat curved, as shown in the lower part of Fig. 9. The curved tongue in this section is similar to the lips in other images; therefore, during the downsampling process of the UNet network, some of its information may be lost, leading to incorrect identification as lips. In RCA-UNet, the combination of the RCBAM-enhanced backbone and MSAG-enhanced skip connections effectively learns the differences between the lips and the curved tongue. This combination greatly preserves detailed information during downsampling, improving the network’s ability to handle such similarity issues in the samples. Overall, RCA-UNet demonstrates better performance in terms of handling detail and similarity issues, which proves that the residual architecture and attention mechanisms play critical roles in enhancing network performance.

In summary, as a part of IF-RCNet, RCA-UNet plays a nonnegligible role in improving the quality of the input information in the proposed IF-RCNet. These findings indicate that RCA-UNet is an indispensable and necessary component of IF-RCNet.

## Discussion

In this study, an IF-RCNet that shows excellent performance was proposed to address the problem of classifying tongue shapes with similar texture or shape features, especially in terms of classifying thin tongues and some normal tongues with special body types. To achieve accurate tongue shape classification, RCBAM with a residual architecture is utilized to enhance the feature perception and extraction ability of the segmentation and classification network. The tongue features extracted by the segmentation network and those extracted by the classification network are combined with the feature fusion method, which allows more tongue features to be acquired without deepening the network structure. In addition, the mixed input approach is combined to expand and process the input data by mixing the tongue texture information obtained from the original input and the segmented tongue contour as the input to the classification network to further improve the tongue shape classification accuracy. The ablation study results clearly demonstrated that, for datasets with different data sizes, the performance of the model with the inclusion or exclusion of feature fusion and mixed inputs exhibited a significant gap, which verified the validity of the proposed method. Moreover, comparison experiments with other tongue classification networks demonstrated that the proposed network outperforms lightweight networks on small sample datasets while being superior to complex networks on challenging datasets. Therefore, the comparison experiments highlighted the superior performance of the proposed IF-RCNet in terms of handling small sample data and similar sample issues. Finally, the effectiveness of the improved tongue segmentation network based on the RCABM and MSAG was analyzed, thus confirming the effectiveness of the residual architecture and attention mechanism-based improvements.

Nevertheless, this study has several limitations. In the future, it will be necessary to develop an AI model specifically for tongue diagnosis to assist physicians in achieving objective and accurate clinical diagnoses. However, challenges such as the difficulty in capturing high-quality tongue images and limitations in the subject population size led to the limited amount of data in this study to support high-performance network training. Additionally, although the investigation results preliminarily validated the potential effectiveness of the proposed method in tongue shape classification tasks, there is still room for further promotion in clinical trials. Considering that deep learning models are often regarded as ‘black box’ models, there is a need for enhanced transparency and interpretability of AI tongue diagnosis models. Further exploration is needed to provide medical professionals with more valuable diagnostic evidence for reference. In the future, more tongue image data will be collected to train high-precision models, and the overall logic of the network will be improved to address interpretability challenges, thereby providing support for the practical application of this model in the health care industry.

## Conclusion

This study investigated a deep learning-based network model for classifying tongue shape, namely, IF-RCNet. The mixed inputs were combined with the feature fusion method to expand the amount of data of the network input and the amount of feature information. An RCBAM and improved convolutional groups were proposed to reinforce the backbone network of the tongue shape classifier, namely, RCA-Net, and that of the tongue segmentation network, namely, RCA-UNet, respectively. To compensate for the upsampling loss, an MSAG was applied to enhance the ability of the skip connection to perceive important features. Thus, the proposed model learned more complex feature information from limited data samples and compensated for the feature loss via the residual architecture and the attention mechanism. An accurate tongue shape classification was achieved, and the adverse effect of insufficient data on network performance degradation was effectively addressed.

A series of rigorous experiments and analyses verified that the proposed network model outperformed most known network models in terms of performance for both tongue shape classification and tongue segmentation tasks. The experimental results illustrated that combining the proposed RCBAM with the improved convolutional groups and the MSAG could simultaneously improve the accuracy of the tongue shape classifier and the performance of the tongue segmentation network. Furthermore, the mixed inputs and the feature fusion method provided an important approach for addressing the limited data and similar samples. These results not only fully confirmed the effectiveness of the proposed IF-RCNet in terms of handling tongue shape classification problems but also highlighted the potential of mixed inputs and feature fusion methods in solving challenges associated with limited medical image data.

## Data Availability

The datasets used and/or analyzed during the current study available from the corresponding author on reasonable request. The code used in this study are publicly available at https://doi.org/10.5281/zenodo.14874334. Other detailed documents can be obtained by contacting 15672806923@163.com.
